# Evaluation of the ability of four EBSL-screening media to detect ESBL-producing *Salmonella* and *Shigella*

**DOI:** 10.1186/s12866-014-0217-3

**Published:** 2014-09-04

**Authors:** Kjersti Sturød, Ulf R Dahle, Einar Sverre Berg, Martin Steinbakk, Astrid L Wester

**Affiliations:** Department of Food-borne Infections, Oslo, Norway; Department of Bacteriology and Immunology, Division of Infectious Disease Control, Norwegian Institute of Public Health, Postbox 4404, Nydalen, 0403 Oslo, Norway

**Keywords:** Antibiotic resistance, ESBL, AmpC, Screening media, *Salmonella*, *Shigella*

## Abstract

**Background:**

The aim of this study was to compare the ability of four commercially available media for screening extended-spectrum beta-lactamase (ESBL) to detect and identify ESBL-producing *Salmonella* and *Shigella* in fecal samples.

A total of 71 *Salmonella-* and 21 *Shigella-*isolates producing ESBL_A_ and/or AmpC, were received at Norwegian Institute of Public Health between 2005 and 2012. The 92 isolates were mixed with fecal specimens and tested on four ESBL screening media; ChromID ESBL (BioMèrieux), Brilliance ESBL (Oxoid), BLSE agar (AES Chemunex) and CHROMagar ESBL (CHROMagar). The BLSE agar is a biplate consisting of two different agars. Brilliance and CHROMagar are supposed to suppress growth of AmpC-producing bacteria while ChromID and BLSE agar are intended to detect both ESBL_A_ and AmpC.

**Results:**

The total sensitivity (ESBL_A_ + AmpC) with 95% confidence intervals after 24 hours of incubation were as follows: ChromID: 95% (90.4-99.6), Brilliance: 93% (87.6-98.4), BLSE agar (Drigalski): 99% (96.9-100), BLSE agar (MacConkey): 99% (96.9-100) and CHROMagar: 85% (77.5-92.5). The BLSE agar identified *Salmonella* and *Shigella* isolates as lactose-negative. The other agars based on chromogenic technology displayed *Salmonella* and *Shigella flexneri* isolates with colorless colonies (as expected). *Shigella sonnei* produced pink colonies, similar to the morphology described for *E. coli*.

**Conclusion:**

All four agar media were reliable in screening fecal samples for ESBL_A_-producing *Salmonella* and *Shigella*. However, only ChromID and BLSE agar gave reliable detection of AmpC-producing isolates. Identification of different bacterial species based on colony colour alone was not accurate for any of the four agars.

## Background

Antimicrobial resistance is an increasing challenge of global proportions [1]. Special emphasis has been put on Gram negative bacteria producing enzymes conferring resistance against beta lactam antibiotics, such as third and fourth generation cephalosporins, monobactams and carbapenems, commonly known as extended spectrum beta-lactamases (ESBLs) [2-4]. ESBLs are associated with higher morbidity and mortality, rising health care costs [5], potential for foodborne transmission [6,7] and asymptomatic carriage [8]. ESBL-producing bacteria most often reside in the intestine of humans and animals, and may thus be difficult to control and eradicate [9,10]. Plasmid mediated ESBL genes can be transferred between different strains of bacteria and between different bacterial species and genera within the Enterobacteriaceae family [11]. Co-resistance to other groups of antibiotics is frequently observed in ESBL-producing organisms, which makes the choice of effective treatment even more limited [12]. In the Nordic countries, recent studies state that the main risk factor for acquiring ESBL-producing bacteria is travel abroad [13-15]. Asymptomatic infections with *Salmonella* and *Shigella* do occur [16,17]. When screening for fecal carriage of ESBL, the methods must ensure reliable detection also of these bacterial species. This is especially important after travel to countries with high prevalence of both ESBL-carrying Enterobacteriaceae as well as high prevalence of these particular pathogens.

The Nordic Committee on Antimicrobial Susceptibility Testing (NordicAST) categorises ESBLs into three broad categories, ESBL_A_, ESBL_M_ and ESBL_CARBA_ according to the classification suggested by Giske et al. [18]. The ESBL_A_- group consists of the classical ESBLs, which are inhibited by clavulanic acid. The group of miscellaneous ESBLs (ESBL_M_) contains plasmid-mediated AmpC and several of the OXA-enzymes. The last category of ESBLs, the ESBL_CARBA_, consists of enzymes that have the ability to inactivate carbapenems.

In this study, Salmonella- and Shigella-isolates classified as ESBL_A_ and/or ESBL_M_ were included according to genotype. All isolates belonging to the ESBL_M_-group were AmpC-genotypes. Several Enterobacteriaceae have chromosomally encoded AmpC-genes but normally the gene expression of these genes is down-regulated [18]. Within genus *Salmonella* the AmpC-gene is not present in the chromosomal genome and AmpC-producing *Salmonella* are thus a product of plasmid mediated AmpC (pAmpC) [19].

To ensure appropriate treatment and to minimize the risk of spread to other patients it is important to detect ESBL-producing strains as early as possible [20]. The fecal carriage rate of ESBL-producing bacteria in healthy populations is increasing, and effective screening-methods for surveillance purposes become increasingly important [8]. Various methods for ESBL-detection have been described, both direct screening on clinical specimens and screening of bacterial isolates [21]. In Norway, clinically relevant strains are routinely tested for the presence of ESBLs, but presently there are guidelines neither on indications nor microbiological strategies for fecal screening. A recent report from the Norwegian Institute of Public Health (NIPH) suggests that patients transferred from hospitals abroad into intensive care units or dialysis units should be screened for fecal carriage of ESBL [22]. However, hospital laboratories may apply different approaches for ESBL screening [23]. In recent years, a variety of ESBL screening media have become commercially available, some which uses chromogenic technology for the direct ESBL-detection in fecal samples. These ESBL screening media are designed to detect and identify ESBL-producing bacteria among the whole Enterobacteriaceae family. The identification of different bacterial species on ESBL screening media is generally based on the enzymatic degradation of different carbohydrates and peptides. *Salmonella*, and some species of *Shigella*, have different sugar degradation profiles than the most predominant cultivatable species within normal fecal flora. So far, most published studies have focused on ESBL-detection in *Escherichia coli* and *Klebsiella spp.* No studies, except one of the manufacturers’, have described how ESBL-producing *Salmonella* or *Shigella* will appear on these four culture media. The aim of this study was therefore to compare commercially available ESBL-screening media to determine their ability to detect and identify of ESBL-producing *Salmonella* and *Shigella* in fecal specimens.

## Methods

The study was carried out at the Norwegian Institute of Public Health (NIPH), Department of Food-borne Infections. This department is the national reference laboratory for food-borne infections and is also responsible for the reporting of antimicrobial resistance in enteropathogenic bacteria at a national level. In 2005, the laboratory initiated screening for ESBL in these bacteria. Since then, nearly 100 ESBL-producing strains of *Salmonella spp*. and *Shigella spp*. have been identified from patients in Norway.

A total of 92 unique isolates *Salmonella* and *Shigella spp.* carrying ESBL_A_ or AmpC genotypes collected between 2005 and 2012 were included based on inhibition zone diameter of ≤ 21 mm against cefpodoxime (Cefpodoxime 10 mcg disc, BBL Sensi-Disc, BD), on Mueller Hinton agar.

### Genotyping of ESBL-producing strains

Prior to the inoculation of the bacteria onto the ESBL agar media, the isolates were characterized by ESBL genotyping. DNA was released from bacterial suspensions of the isolates by heat treatment (95°C, 5 min) and first tested in three ESBL_A_ PCR assays [24]. As a part of this study, and without changing the primer sequences these ESBL_A_ assays were converted into real-time PCR format to enable DNA melt analysis. The real-time PCR adaption of the protocol was achieved through use of the double-strand-DNA-specific fluorescent reporter dye SYTO®9 (Invitrogen), the ammonium sulfate/Tris-based PCR buffer IV (ABgene®) and Platinum Taq DNA polymerase (Invitrogen) [25,26]. The amplification and the subsequent DNA melting of the amplification products were done in a StepOnePlus™ Real-Time PCR instrument (Life Technologies™). The three ESBL_A_ real-time PCR assays indicated presence of *bla*_TEM_, *bla*_SHV_, and *bla*_CTX-M_ in the samples. In addition, the bacterial DNA was also tested in two ESBL_M_ triplex PCR assays by use of the published primers and primer combinations as *bla*_CIT_/*bla*_MOX_/*bla*_FOX_ and *bla*_DHA_/*bla*_ACC_/*bla*_EBC_ [27]. Without change of the AmpC primer sequences, the reaction conditions of the two triplex assays were modified, as for the above ESBL_A_ assays, to SYTO®9-based real-time PCR. The DNA melt analysis discriminated the various products of the two AmpC triplex PCR assays. All of the ESBL-positive PCR products were subjected to bidirectional DNA sequencing to confirm the real-time results. Finally the ESBLA and AmpC isolates were sub-typed by comparison to a BioEdit database made from sequences deposited in GenBank (http://blast.ncbi.nlm.nih.gov/Blast.cgi) according to the beta-lactamase classification in the Lahey database. (http://www.lahey.org/Studies/) [28]. Four of the 92 isolates originally included in the study based on the cefpodoxime screening test (disc diffusion test) were found to be ESBL-negative by PCR. One of the PCR-positive isolates did not grow on subculture. These five strains were excluded from the study, resulting in a total of 87 eligible isolates. Of the 87 isolates included in the study there were 17 isolates of *Shigella sonnei*, two isolates of *Shigella flexneri*, 18 isolates of *Salmonella* Typhimurium, 12 isolates of *S.* Stanley, seven isolates of *S.* Concord, five isolates of *S.* Enteritidis and 16 isolates of other non-Typhoid *Salmonella*.

### Fecal samples

To mimic fecal samples, we followed the same procedure as has been applied in the Norwegian external quality control program, organized by the NIPH. A fecal suspension from a healthy person was prepared, after controlling for the absence of *Salmonella* and *Shigella*. The donor fecal material was diluted (approximately 1:5) with isotonic NaCl solution (0.9%). A part of the suspension was heated (80°C, 1 hour) to prevent bacterial overgrowth from intestinal flora on the ESBL screening agars. For each of the 87 samples, 0.9 ml of the heat-treated fecal suspension and 0.1 ml of the non-heated suspension were mixed with 1 ml of Cary-Blair-medium. Table 1 presents the procedure applied to standardize the quantity of ESBL-producing bacteria inoculated on the screening agars. Pure culture of each of the ESBL-producing bacteria was suspended in 0.9% NaCl-solution. The optical density (OD) was then adjusted to 0.40, measured with a spectrophotometer (Helios Epsilon from Thermo Scientific). 30 μl of each pure-culture suspension containing ESBL-producing isolates was added to the fecal suspensions. In addition, to mimic normal growth, non-ESBL *E. coli* (50–200 μl with an OD of 0.40) isolated from the donor feces was added to the suspensions from a pure culture. One droplet (50 μl, equivalent to ~8x10^4^ CFU of ESBL-positive culture) of each of the 87 spiked fecal suspensions were spread onto each of the four ESBL screening agars, and on lactose-agar and XLD-agar as controls. In addition, pure culture from the ESBL-carrying isolates was inoculated onto the four screening agars to ensure that all the ESBL-carrying bacteria did grow on all four media and to facilitate the reading of the corresponding agars inoculated with the fecal specimens. All screening agars were incubated in ambient air at 37°C. After 24 hours incubation, the degree of growth was graded from 0; no growth, to 3; excellent growth.

**Table 1:** **Content of the fecal suspension**

**Fecal suspension** ^**1**^	
**900 μL**	Heat treated feces (non-ESBL)
**100 μL**	Non-heated feces (non-ESBL)
**1000 μL**	Cary Blair-medium
**30 μL**	Pure culture (ESBL) OD: 0.4 (1,2x10^8^/mL)
**50-200 μL**	Non-ESBL *E. coli* OD: 0.4 (1.2x10^8^/mL)
**~2100 μL**	

^1^50 μL from this suspension was inoculated on each screening agar.

The preparation, inoculation and interpretation of the culture media were manually performed.

### ESBL screening media tested

Four commercially available selective media designed to detect ESBL-producing bacteria directly from clinical specimens were compared. ChromID ESBL (BioMèrieux, Lyon, France), Brilliance ESBL (Oxoid, Basingstoke, United Kingdom) BLSE agar (AES Laboratoire, Combourg, France) and CHROMagar ESBL (CHROMagar, Paris, France) are all selective agar media commercially available in Norway. The BLSE agar is a bi-plate made of two different non-chromogenic selective media, MacConkey agar and Drigalski agar.

According to the product information provided by the manufacturers, all four agars contain an extended-spectrum cephalosporin, in combination with other antibacterial agents to inhibit growth of non-ESBL Enterobacteriaceae. Both ChromID ESBL and Brilliance ESBL media are supplemented with cefpodoxime in addition to an undeclared mixture of antibacterial agents. The cefpodoxime concentration in these two plates is not given. The BLSE MacConkey agar is supplemented with ceftazidime (2 mg/L) while the BLSE Drigalski agar is supplemented with cefotaxime (1.5 mg/L). CHROMagar is supplemented with an unknown mixture of antibacterial agents. Two of the screening agars, Brilliance ESBL and CHROMagar ESBL, are expected to suppress growth of AmpC-producing bacteria while ChromID ESBL and BLSE agar are designed to select also for AmpC-positive bacteria.

ChromID ESBL, Brilliance ESBL and CHROMagar contain different chromogens which target different enzymes within different bacterial species, resulting in coloured colonies making identification easier. The chromogenic substrates differ between the three agars, but all of them seem to target β-galactosidase and/or β-glucuronidase (*Klebsiella*, *Serratia*, *Enterobacter and Citrobacter,* commonly known as the KSEC-group, and *E. coli*) and deaminase (*Proteus*, *Providencia* and *Morganella*). According to the manufacturers’ information, *E. coli* will appear pink on ChromID and CHROMagar, and pink or blue on the Brilliance agar. Furthermore, the KSEC-group will appear green on ChromID and Brilliance agar, while on CHROMagar the KSEC-group will appear blue. *Proteus*, *Providencia* and *Morganella* will appear brown on all three chromogenic agars according to the product information.

It is known that *Shigella sonnei* produces β-galactosidase and β-glucuronidase and will thus appear like *E. coli* on the chromogenic agars [29]. In comparison, neither *Shigella flexneri* nor *Salmonella* generally produce any of these enzymes and will consequently appear with colourless colonies [29-31]. The appearance of *Salmonella* and *Shigella* is, however, not stated by the manufacturers, with the exception of the Brilliance ESBL agar. This manufacturer describes that *Salmonella* will appear colorless.

The BLSE agar does not contain a specific chromogenic substrate, but has the ability to detect and differentiate ESBL-positive Enterobacteriaceae and other multiresistant Gram negative bacilli based on their ability to ferment lactose. The MacConkey agar and the Drigalski agar both contain pH-indicators which differentiates lactose-positive and lactose-negative bacteria based on the color of the agar and the colonies. Most species within the *Salmonella* and *Shigella* genera do not have the ability to ferment lactose. However, *Shigella sonnei* may ferment lactose, but only after extended incubation [31].

ChromID ESBL, Brilliance ESBL and BLSE agar are available as “ready to use” plates from the producers, while CHROMagar ESBL is sold as a powder base.

### Statistical analyses

The calculation of the sensitivity for detecting ESBL-carrying isolates for each screening agar was based on a total of 87 isolates, 51 isolates carrying ESBL_A_ genotypes and 36 carrying AmpC genotypes. The single isolate which was both ESBL_A_ - and AmpC positive was counted as an AmpC in the statistical analysis. For each agar plate the total sensitivity was calculated (ESBL_A_ + AmpC) (n = 87), as well as the sensitivity for ESBL_A_ and AmpC alone (n = 51 and n = 36, respectively). A 95% confidence interval (95% CI) for each value was manually calculated using binomial proportions’ confidence interval.

## Results

**Figure 1:**
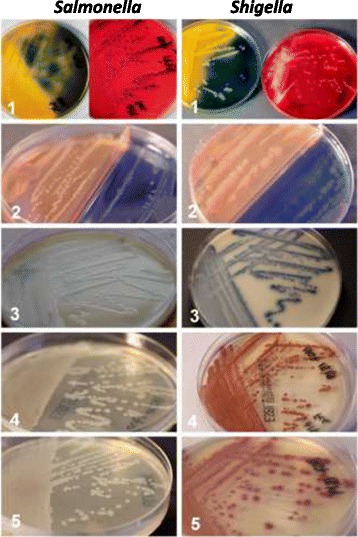
**Picture of normal growth of** ***Salmonella*** **(left) and** ***Shigella sonnei*** **(right) with ESBL genotypes.** All ESBL positive isolates were mixed with a fecal suspension controlled for the absence of *Salmonella, Shigella* and any other ESBL-producing bacteria, before being inoculated onto the screening agars. The Lactose and XLD agars (top) were used as controls. a = *Salmonella*, b = *Shigella sonnei*, 1 = Lactose + XLD (control agars), 2 = BLSE agar, 3 = Brilliance ESBL, 4 = ChromID ESBL, 5 = CHROMagar ESBL.

The ESBL genotyping results are shown in Tables 2 and 3. The genotypic characterisation enabled prediction of growth and color of the colonies growing on the various media. The expected outcome was compared with the observed results. The expected colony colours for *Salmonella spp.* and *Shigella sonnei* on each ESBL screening agar are shown in Figure 1. The grading of growth for the 87 isolates is presented in Tables 4 and 5, respectively. The calculated sensitivity is presented in Table 6.

**Table 2:** **Distribution of ESBL-genes in the 87 isolates**

	**ESBL**_**A**_			**ESBL**_**A**_ **+ AmpC**	**AmpC**		**Total**
	**CTX-M**	**SHV-12**	**CTX-M −15 + SHV-12**	**TEM-63 + CMY-2**	**CMY-2**	**DHA-1**	
***Salmonella***	26	3	4	1	33	1	68
***Shigella***	18	0	0	0	1	0	19
**Total**	44	3	4	1	34	1	87

**Table 3:** **Genotypes within the CTX-M-isolates**

	***Salmonella***	***Shigella***
CTX-M-1	1	0
CTX-M-3	0	1
CTX-M 3/22	1	0
CTX-M-9	1	0
CTX-M 14/17/18	7	1
CTX-M 15	16	15
CTX-M-27	0	1
	26	18

**Table 4:** **Grading of growth of 68 ESBL**_**A**_ **- and/or AmpC-producing** ***Salmonella*** **isolates (n=68)**

**Growth**	**Excellent**		**Good**		**Poor**		**No growth**	
	**ESBL**_**A**_	**AmpC**	**ESBL**_**A**_	**AmpC**	**ESBL**_**A**_	**AmpC**	**ESBL**_**A**_	**AmpC**
**Brilliance ESBL**	31	9	1	5	1	17		4
BLSE agar* – Drigalski	31	35	1				1	
BLSE agar* – Mac Conkey	31	34		1	1		1	
**CHROMagar ESBL**	32	4	1	4		14		13
**ChromID ESBL**	33	12		16		4		3

All ESBL-producing isolates were mixed with a fecal suspension controlled for the absence of *Salmonella*, *Shigella* and any other ESBL-producing bacteria, before being inoculated on the screening agars.

*BLSE agar is a biplate consisting of one half of Drigalski agar and one half of MacConkey agar.

**Table 5:** **Grading of growth, of 19 ESBL**_**A**_ **- or AmpC-producing** ***Shigella*** **isolates (n=19)**

**Growth**	**Excellent**		**Good**		**Poor**		**No growth**	
	**ESBL**_**A**_	**AmpC**	**ESBL**_**A**_	**AmpC**	**ESBL**_**A**_	**AmpC**	**ESBL**_**A**_	**AmpC**
**Brilliance ESBL agar**	18							1
BLSE agar* – Drigalski	16	1	2					
BLSE agar* – Mac Conkey	15	1	3					
**CHROMagar ESBL**	18					1		
**ChromID ESBL**	17		1					1

All ESBL-producing isolates were mixed with a fecal suspension controlled for the absence of Salmonella, Shigella and any other ESBL-producing bacteria, before being inoculated on the screening agars.

*BLSE agar is a biplate consisting of one half of Drigalski agar and one half of MacConkey agar.

**Table 6:** **A comparison of the expected and observed result, colour of colonies and sensitivity**

	**ChromID ESBL**	**Brilliance ESBL**	**Drigalski (BLSE agar)**	**MacConkey (BLSE agar)**	**CHROMagar ESBL**
**Observed /Expected ESBL**_**A**_ **-positive**	51/51	51/51	50/51	50/51	51/51
**Observed/Expected AmpC-positive**	32/36	31/0	36/36	36/36	23/0
**Expected colour of colonies**	Colourless	Colourless	Blue	White	Colourless
**Colour of** ***Salmonella*** **colonies**	Colourless (n = 62) Pink (n = 3)	Colourless (n = 61) Pink (n = 3)	Blue	Pale pink	Colourless
**Colour of** ***Shigella sonnei*** **colonies**	Pink	Blue	Blue	Pale pink	Pink
**Colour of** ***Shigella flexneri*** **colonies**	Colourless	Colourless	Blue	Pale pink	Colourless
Sensitivity (95% CI*)	95% (90.4 - 99.6)	93% (87.6 - 98.4)	99% (96.9 - 100)	99% (96.9 - 100)	85% (77.5 - 92.5)
**Sensitivity ESBL**_**A**_ (95% CI*)	100%	100%	98% (94.2 - 100)	98% (94.2 - 100)	100%
Sensitivity AmpC (95% CI*)	89% (78.8 - 99.2)	83% (70.7 - 95.3)	100%	100%	64% (48.3 - 79.7)

A total of 87 ESBL-producing isolates (51 = ESBL_A_, 36 = AmpC) were inoculated on the four screening agars. BLSE agar is a biplate consisting of two different agars; Drigalski agar and MacConkey agar. The isolates were mixed with a fecal suspension before inoculation. The expected results are estimated by the manufacturer’s product information.

*CI = 95% Confidence interval.

### ChromID ESBL

All of the 87 spiked fecal samples were expected to be detected on ChromID ESBL agar as colourless colonies. All of the 51 isolates carrying ESBL_A_ genotypes, but only 32 of the 36 AmpC isolates were detected (Table 6). The four AmpC isolates that did not grow on ChromID, all carried *bla*_CMY-2._ Three *Salmonella-*isolates made pink colonies while the rest of the growing *Salmonella* isolates (n=62) produced colourless colonies. *Shigella sonnei* (n=16) and *Shigella flexneri* isolates (n=2) produced pink and colourless colonies, respectively. The total sensitivity of ChromID ESBL was 95% (95% CI 90.4-99.6%), the sensitivity for ESBL_A_ was 100%, and the sensitivity for AmpC was 89% (95% CI 78.8-99.2). ChromID ESBL had overall higher graded growth with ESBL_A_-positive strains than AmpC-positive (Tables 4 and 5).

### Brilliance ESBL

The expected results for the Brilliance ESBL agar were that all 51 isolates carrying ESBL_A_ genotypes would grow and appear as colourless colonies, in contrast to the 36 AmpC isolates that should be suppressed. The observed results showed that all the 51 ESBL_A_-positive isolates were detected, while 30 of the 36 AmpC isolates were not suppressed and did grow (Table 6). The growth of these 30 AmpC-isolates was generally scored lower than the ESBL_A_-isolates. Three *Salmonella* isolates produced pink colonies while the rest of the *Salmonella* isolates (n=61) detected, produced colourless colonies. *Shigella sonnei* (n=16) and *Shigella flexneri* (n=2) isolates produced blue and colourless colonies, respectively. The total sensitivity for ESBL detection of Brilliance ESBL agar was 93% (9% CI 87.6-98.4%), the sensitivity for ESBL_A_ was 100% and the sensitivity for AmpC was 83% (95% CI 70.7-95.3%).

### BLSE agar

The expected results for CHROMagar ESBL were that all 51 isolates with ESBL_A_ genotypes would be detected with colourless colonies, while the growth of the 36 AmpC isolates would be inhibited. The observed results were that CHROMagar ESBL detected all the 51 ESBL_A_ isolates, but 23 of the 36 AmpC isolates were not inhibited (Table 6). The growth of these 23 AmpC-isolates was generally graded lower than the ESBL_A_-isolates. All detected isolates of *Salmonella* (n=55) and *Shigella flexneri* (n=17) produced colourless colonies while *Shigella sonnei* (n = 2) produced pink colonies. The total sensitivity for ESBL detection of CHROMagar was 85% (95% CI 77.5-92.5%), the sensitivity for ESBL_A_ detection was 100% and the sensitivity for AmpC was 64% (95% CI 48.3-79.7%).

### CHROMagar ESBL

The expected results for CHROMagar ESBL were that all 51 isolates with ESBL_A_ genotypes would be detected with colourless colonies, while the growth of the 36 AmpC isolates would be inhibited. The observed results were that CHROMagar ESBL detected all the 51 ESBL_A_ isolates, but 23 of the 36 AmpC isolates were not inhibited (Table 6). The growth of these 23 AmpC-isolates was generally graded lower than the ESBL_A_-isolates. All detected isolates of *Salmonella* (n = 55) and *Shigella flexneri* (n = 17) produced colourless colonies while *Shigella sonnei* (n = 2) produced pink colonies. The total sensitivity for ESBL detection of CHROMagar was 85% (95% CI 77.5-92.5%), the sensitivity for ESBL_A_ detection was 100% and the sensitivity for AmpC was 64% (95% CI 48.3-79.7%).

## Discussion

To the best of our knowledge, our study is the first comparing commercially available ESBL screening media, for direct screening of ESBL-carrying *Salmonella* and *Shigella* in fecal samples. One study conducted by Kocagöz et al. [32] evaluated a novel chromogenic medium, Quicolor E&S agar, for the detection of ESBL-producing *Salmonella spp*. However, Quicolor E&S seems not to be designed for the direct screening of clinical samples [32]. Since other Enterobacteriaceae and non-Enterobacteriaceae carrying ESBL have been evaluated in other studies, we did not focus on these bacteria [33-36].

All bacterial isolates in the study had reduced sensitivity against cefpodoxime, and carried genotypes conferring ESBL_A_ or AmpC phenotypes; hence this study was not designed to reveal false positive results. The specificities of the four screening agars have been documented in previous studies focusing on the ability to detect ESBL-producing bacteria within the Enterobacteriaceae family. These studies included none or just a few *Salmonella* isolates, and the specificity varied greatly. ChromID ESBL agar was included in most of the studies, and the specificity ranged from 72.9% - 94.9% [33-36]. The specificity of the Brilliance agar ranged from 57.9%– 95.1% [33,34,36], and for BLSE agar the specificity ranged from 60.8-85.0% [34,35]. CHROMagar ESBL has been evaluated by Grohs et al. only, with a reported specificity of 72.3% [33]. However, some of the previous studies seem to have included ESBL-producing non-Enterobacteriaceae isolates as test positives, while other studies only included ESBL-producing isolates within the Enterobacteriaceae family. This difference may explain the apparent great variations in specificities reported.

The frequency of human infection with *Salmonella* and *Shigella* in Norway is relatively low. Consequently, to gain proper statistical power in a real-life study evaluating screening plates for ESBL-positive strains of these two genera would be time consuming. We therefore chose to use a suspension of a normal fecal sample spiked with the ESBL- positive isolates. The quantity of ESBL-positive bacteria in the fecal samples is known to be a factor of the sensitivity of the screening agars [37]. In genuine fecal samples the quantity of bacteria varies, but in this study we spiked the same quantity of bacteria in all samples.

*Salmonella* are normally lactose negative and produce neither β-galactosidase nor β-glucuronidase. Consequently, colonies of *Salmonella* appeared colourless on agarplates that use these enzymes in the chromogenic reactions. *Shigella sonnei* is both β-glucuronidase and β-galactosidase-positive and appeared much like *E. coli* on these screening agars. Therefore direct differentiation of *Shigella sonnei* and *E. coli* is difficult. However, none of the manufacturers mention this similarity in their product information. On the other hand, *Shigella flexneri* does not express these enzymes, and will not appear like *E. coli* on the screening agars. This was confirmed in our testing. Obviously, testing only two *Shigella flexneri* isolates is insufficient to give a statistically reliable result. Three *Salmonella* isolates of different serovars had pink colonies on both ChromID and Brilliance agars, whereas the rest of the *Salmonella* isolates had colorless colonies. It is necessary for the pink color formation that the bacteria express β-glucuronidase, which is described that some *Salmonella* bacteria actually do [38]. The color-based identification was non-specific and comparable to expected results from using a non-chromogenic agar with the same antibacterial supplements. Consequently, any growth on the ESBL screening agars, regardless of manufacturer, needs to be further confirmed by phenotypic or genotypic analyses. The BLSE agar which distinguishes the bacterial species according to their lactose fermentation capability separates *E. coli* and *Klebsiella* from *Salmonella* and *Shigella*. The manufacturers of Brilliance agar and CHROMagar claim that their screening agars inhibit the growth of AmpC-positive bacteria. This may limit the use of these growth media since plasmid-mediated AmpC is increasing in prevalence. On the other hand, specific ESBL_A_ detection can be useful in the clinical setting of outbreak with ESBL_A_ carrying strains. In our study, both Brilliance agar and CHROMagar did not inhibit growth of AmpC-positive strains in the way that the producers claim they would. However, the majority of the AmpC-positive isolates included in this study belonged to the CMY-2 genotype and this result may not be generalizable to other genotypes. Our results also showed that these media did not support growth of AmpC-positive isolates as well as they did for ESBL_A_-positive isolates indicating that the growth was suppressed rather than totally inhibited. This observation may be of importance in real fecal samples where mixed bacterial flora may lead to overgrow of partly suppressed slow growing AmpC-positive isolates. However, in this study when interpreting the growth on the agars, any growth was considered positive.

There was no pronounced difference between different serovars in the material. The isolates which were inhibited consisted of nine different *Salmonella* serovars and one *Shigella sonnei*. Other isolates belonging to the same serovars as the inhibited isolates showed excellent growth on all agars, except *S*. Cholerasuis which were inhibited on CHROMagar, ChromID and Brilliance agar. There was only one *S.* Cholerasuis included in this study and no conclusion can be made from this isolate alone.

We find that the sensitivity for ESBL detection of ChromID agar and BLSE agar was satisfying, and that both agars enabled the detection of almost every ESBL-positive isolate, regardless of ESBL genotype or serovar/serogroup. The Drigalski part of the BLSE agar was the only agar that showed both *Salmonella* and *Shigella* isolates with colored colonies. The blue color indicated that the bacteria were lactose-negative or that the lactose fermentation was dependent of an extended incubation. The blue colour enabled differentiation of *Salmonella* and *Shigella* from the most usual ESBL-producing *E. coli* and *Klebsiella spp.* The blue colour does not differentiate the isolate from multi resistant Gram negative bacilli other than Enterobacteriaceae, such as *Pseudomonas aeruginosa*, *Acinetobacter* and *Stenotrophomonas maltophilia*.

## Conclusions

The main conclusion of this study is that the method of screening fecal samples by the use of selective agar plates was easy to perform and the four agars detected the presence of ESBL-carrying bacteria in overnight cultures. All four agar media appeared reliable for screening for both *Salmonella* and *Shigella* with ESBL_A_ genotypes from fecal samples. However, only ChromID agar and BLSE agar were reliable in detecting isolates with AmpC.

Furthermore, the BLSE agar had the highest sensitivity and was the only agar which differentiated *E. coli* and *Klebsiella* from *Salmonella* and *Shigella* by the colour of the colonies. The three other agars differentiated *E. coli* and *Klebsiella* from *Salmonella* and *Shigella flexneri* by the colourless colonies of *Salmonella* and *Shigella flexneri* and the coloured colonies of *E. coli* and *Klebsiella*. These three agars did not enable differentiation between *E. coli* and *Shigella sonnei.* The BLSE agar and the ChromID were both good alternatives for screening of fecal specimens with ESBL positive *Salmonella* or *Shigella*. The BLSE agar had the highest sensitivity, while ChromID had fairly good sensitivity. ChromID had a higher sensitivity for ESBL_A_-than AmpC bacteria, while BLSE agar was equally sensitive to both ESBL_A_- and AmpC bacteria. Because detection of ESBL-carrying Salmonella and *Shigella* is highly important both in clinical settings and for surveillance purposes, the strengths and weaknesses hereby reported should be taken into consideration when using any of these four commercially ESBL screening agars.
